# Supplementary material for: Seasonal differences in predation risk among seagrass epifauna species stabilize community‐level predation over time
Source: Ecology. 2026 May 4;107:e70401. doi: 10.1002/ecy.70401 (PMC13137078; doi:10.1002/ecy.70401)
Supplement: Supplementary file 3 — Video S1 Metadata. [file ECY-107-e70401-s003.pdf]

Video S1 Metadata

Seasonal differences in predation risk among seagrass epifauna species stabilize community-level predation over time. Claire E. Murphy & John J. Stachowicz. *Ecology.*

Video S1. A shiner perch (*Cymatogaster aggregate*) can be seen eating a tethered *Ampithoe valida* before swimming off at a site with a high amount of eelgrass cover (Blake's Landing). Video was taken by Claire Murphy.
